# Determination of Aluminum in Precipitation Hardening Stainless Steel and High Temperature Alloys

**DOI:** 10.6028/jres.064A.019

**Published:** 1960-04-01

**Authors:** Lawrence A. Machlan, John L. Hague, Edward J. Meros

## Abstract

A procedure is described for the determination of aluminum in high temperature alloys. Aluminum is selectively precipitated with 8-hydroxyquinoline from an ammoniacal solution of the alloy containing citrate and cyanide as complexing agents. The precipitate is ignited under oxalic acid, the oxides fused, and dissolved in acid. A caustic precipitation is made, an aliquot of the filtrate treated with hydrogen peroxide, and the aluminum precipitated with 8-hydroxyquinoline. The aluminum hydroxyquinolate is filtered on a fritted-glass crucible, dried, and weighed.

## 1. Introduction

One of the interesting developments in metallurgy during the last few years has been the emergence of high temperature alloys and precipitation hardening stainless steels as an important group of alloys. Many, if not most, contain aluminum as an alloying addition. All contain chromium, and varying amounts of iron, cobalt, and nickel as major constituents. Titanium, zirconium, and molybdenum are frequently present as alloying additions; less frequently, niobium and tungsten may also be used.

This combination of elements makes the determination of aluminum by chemical methods a vexing one. An embarrassingly large number of choices is available when it comes to choosing separative and determinative steps for an analysis, and most can be made to work under proper conditions; in fact, it has been said with some justification that there are almost as many methods for determining aluminum as there are analysts willing to write them. It is beyond the scope of this paper to present a comprehensive survey. The method which follows is a useful combination of steps that has been used over a period of years in the Bureau and found to have better than average reliability.

The sample, usually 2 g, is dissolved in a nitric- hydrochloric acid mixture. Citric acid is added as a complexing agent, and the solution made ammoniacal. Sodium cyanide is added to form complexes with nickel, iron, copper, and cobalt, and the aluminum precipitated by the addition of 8-hydroxyquinoline. The precipitate is filtered, ignited after the addition of oxalic acid, and the ignited residue treated with sulfuric and hydrofluoric acids to eliminate silica. The residue is fused in bisulfate, leached in hydrochloric acid, the solution partially neutralized, and a sodium hydroxide precipitation made. An aliquot of the filtrate (usually representing 1 g of sample) is treated with hydrogen peroxide and sodium cyanide, and the aluminum again precipitated with 8-hydroxyquinoline, filtered, dried, and weighed.

The separation of aluminum with 8-hydroxyquinoline in the presence of cyanide was originally described by Heczko [3]^1^ in 1934. In conjunction with a sulfide separation to remove manganese, it has been used for some years as a simple routine procedure for the determination of aluminum in nitriding steels. Unfortunately the method lacks specificity, as zirconium and titanium, to name two elements, are serious interferences. Tartaric acid is usually used [6, 7] to minimize the co-precipitation of chromium, but in our experience citric acid is somewhat better in this respect. From experiments covering a range of concentrations, the amounts of citric acid and sodium cyanide were chosen as about the minimum quantities required to consistently give a reasonably clean separation of aluminum from chromium, nickel, cobalt, and iron.

A discussion of the sodium hydroxide separation is almost redundant. Much pertinent information will be found in an article by Bright and Fowler [1]. The separation serves to remove manganese, titanium, zirconium, and magnesium among others [4], as well as the remainder of the major elements. Since the latter are largely eliminated in the previous step, the precipitation is run under controlled conditions in the presence of a small addition of iron. Stainless steel beakers and polyethylene beakers and funnels can be used, and obviate the use of expensive platinum, or the use of glassware in contact with strongly alkaline solutions.

Hydrogen peroxide is added to the acidified aliquot to complex any residual titanium, niobium, vanadium, molybdenum, etc. The aluminum is precipitated with 8-hydroxyquinoline, filtered on a glass frit, dried, and weighed. This separation was described by Lundell and Knowles [5] in 1929, and is a convenient method for removing those elements which form peroxide complexes.

The method has proved useful in the determination of aluminum in the 0.5 to 3.0 percent range in a variety of alloys. Experiments on synthetic mixtures indicate the accuracy to be of the order of one percent or better of the amount of aluminum present. The tendency of the final precipitate of the aluminum quinolate to run slightly (about 1%) high almost exactly compensates the small absorption and solubility losses. The method requires more elapsed time than some possible alternatives, due primarily to the steps involving ignition and treatment with sulfuric and hydrofluoric acids. Since these steps do not require much attention, the “working time” is usually about a day for a set of six determinations.

## 2. Reagents

### Citric acid solution (500g/liter)

Dissolve 500 g of citric acid monohydrate in 500 ml of diluted sulfuric acid (l+49)^2^, dilute to 1 liter with water, and filter to remove insoluble material.

### 8-Hydroxyquinoline solution (75 g/liter of ethanol)

Dissolve 75 g of 8-hydroxyquinoline in 900 ml of ethyl alcohol, dilute to 1 liter with ethyl alcohol, and filter.

### Cyanide wash-solution

Dissolve 20 g of ammonium chloride, 20 g of ammonium citrate, and 20 g of sodium cyanide in 900 ml of water containing 25 ml of ammonium hydroxide, and dilute to 1 liter with water.

### Iron solution (20 mg/ml)

Dissolve 10 g of iron containing a negligible quantity of aluminum in 200 ml of diluted hydrochloric acid (1 + 3), cautiously oxidize with 8 to 10 ml of nitric acid, cool, and dilute to 500 ml with water.

### Sodium hydroxide solution (300 g/liter)

Transfer 700 ml of water and 300 g of sodium hydroxide to a polyethylene bottle, dilute to 1 liter with water, and mix well.

## 3. Procedure

Transfer 2 g of the sample to a 600-ml beaker, cover, and add 40 ml of diluted aqua regia (1 + 1) (3 parts of hydrochloric acid and 1 part of nitric acid diluted with an equal volume of water). Warm the solution on a steam bath until the sample dissolves. Add 40 ml of citric acid solution and adjust the volume of the solution with water to 250 ml. Add ammonium hydroxide until the solution is but slightly acid, add 1 g of hydroxylamine hydrochloride, neutralize to litmus paper with ammonium hydroxide, and add 10 ml in excess. Add 15 g of sodium cyanide,^3^ heat to boiling, and boil 1 to 2 min. Add 25 ml or a sufficient^4^ amount of 8-hydroxyquinoline solution slowly while stirring the solution vigorously. Stir the solution vigorously (best done with a mechanical stirrer) for 15 min and allow the solution to stand 15 min on the edge of the steam bath. Add paper pulp, stir the solution well to distribute the pulp, and cool to room temperature.

Filter the solution through a double thickness of close-texture filter paper fitted to a 7-cm Büchner funnel and precoated with a little filter pulp. Transfer the precipitate to the funnel, and wash 5 to 6 times with the cyanide wash solution,^5^ and 2 to 3 times with water. Transfer the paper and precipitate to a platinum crucible, cover the precipitate with 5 g of oxalic acid, place the crucible in a cold muffle, and slowly ignite to 600 to 700° C. Add 1 to 2 ml of diluted sulfuric acid (1 + 3) and 10 to 15 ml of hydrofluoric acid to the crucible, and remove the hydrofluoric and sulfuric acids by heating on an air or sand bath. Add 1 to 2 g of potassium pyrosulfate to the crucible and fuse the salt to dissolve the oxides. Transfer the crucible and fusion to a 400-ml beaker and dissolve the fusion in 100 ml of warm diluted hydrochloric acid (1 + 5). Remove and wash the crucible with diluted hydrochloric acid (1 + 5). Add 5 ml of iron solution, nearly neutralize (as indicated by the slow redissolving of the iron precipitate) with sodium hydroxide solution (300 g/liter). Transfer 140 ml of water and 70 ml of sodium hydroxide solution to a weighed^6^ 1-liter stainless steel beaker. Heat the sodium hydroxide solution to boiling, add the sample solution slowly with good stirring, and boil for 1 to 2 min. Cool the solution to room temperature, dilute with water to 500 g, stir thoroughly, and allow the precipitate to settle for a few minutes. Filter through a close-texture paper fitted to a polyethylene funnel. Discard the first 20 to 30 ml and collect approximately half of the solution in a weighed 600-ml polyethylene beaker. Reweigh the beaker to obtain the weight of the aliquot.

Transfer the filtered solution to a 600-ml glass beaker containing 25 ml of hydrochloric acid and 2 ml of citric acid solution. Neutralize the solution with ammonium hydroxide and add 10 ml in excess. Add 1 g of sodium cyanide and heat the solution to 55° to 60° C. Add 1 ml of hydrogen peroxide (30%) to the warm solution, and slowly add sufficient^7^ 8-hydroxyquinoline solution while stirring the solution vigorously. Stir the solution vigorously for 15 min and allow the solution to stand 15 min on the edge of the steam bath. Cool the solution to room temperature, filter through a weighed close-fritted glass crucible, and wash the precipitate 10 to 12 times with water. Dry the crucible and precipitate for 1½ hr at 115° C, cool, and weigh.

## 4. Discussion and Results

**Figure 1:**
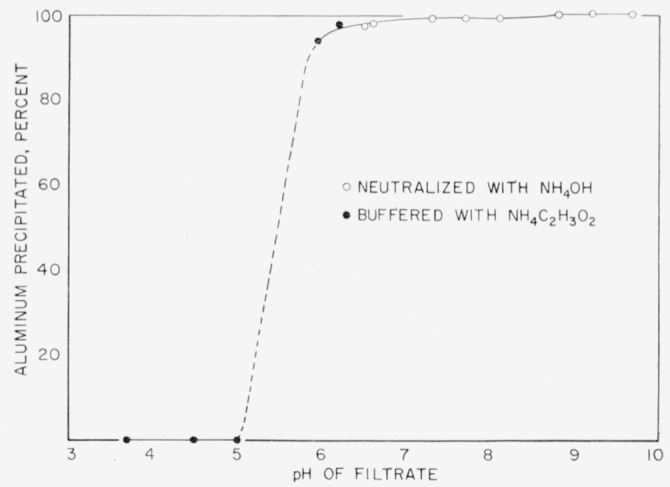
Effect of p*H* on the precipitation of aluminum with 8-hydroxyquinoline in the presence of citric acid.

The precipitation of aluminum with 8-hydroxyquinoline is restricted to a somewhat narrower *pH* range in the presence of citric acid than is the usual case using tartaric or acetic acid. A *p*H-precipitation curve for aluminum hydroxyquinolate in the presence of citric acid is given in figure 1. The recoveries involve approximately 10 mg of aluminum in a volume of 150 ml, in the presence of 1 g of citric acid. Adjustment of the *pH* was made with ammonium hydroxide and acetic acid as indicated, and the *pH* was determined on a portion of the filtrate at room temperature. The first filtrate for samples run according to the recommended procedure was usually in the *pH* range of 9.2 to 9.4, which is a satisfactory point for complete precipitation of aluminum and nearly complete solution of molybdenum and tungsten [2]. Nickel, cobalt, iron, and chromium, the major elements, are also largely eliminated in the first precipitation.

The values obtained in table 1 show that aluminum in amounts from 10 to 100 mg (corresponding to 0.5 to 5% on the aliquot used) can be determined by the recommended procedure with an error of the order of 1 percent of the amount present. The values obtained for aluminum in amounts below 10 mg (5 mg or less in the final aliquot) almost always indicated a negative bias, and the method as written is not satisfactory for alloys containing less than 0.5 percent aluminum. These data also show that titanium at the 2-percent level is not an interfering constituent.

The data in table 2 demonstrate that niobium, tantalum, molybdenum, tungsten, vanadium, pliosphorus, tin, and zirconium in amounts apt to be encountered in these alloys do not interfere. The use of NBS Standard Samples 168 and 169 as base material for the preparation of synthetic mixtures show that major amounts of nickel, cobalt and chromium do not interfere. A few values obtained on several high temperature alloys are tabulated in table 3, to show the agreement which can be expected on replicate determinations. The single run on the steel standard 106a is given to show that major amounts of iron do not interfere, but is not otherwise pertinent because simpler methods are available for this type of alloy.

## Figures and Tables

**Table 1:** Results of determinations of aluminum by the recommended procedure in various synthetic mixtures

NBS Standard Sample 169, Ni 77, Cr 20, A1 0.095					
Weight of sample	Other element added	Aluminum			

		Added	Present	Found	Difference
					
*g*	*mg*	*mg*	*mg*	*mg*	*mg*
2	40 Ti	………	1.9	1.4	−0.5
2	40 Ti	5.0	6.9	6.5	−.4
2	40 Ti	5.0	6.9	6.7	−.2
2	40 Ti	10.0	11.9	11.7	−.2
2	40 Ti	10.0	11.9	11.8	−.1
2	40 Ti	20.0	21.9	22.2	+.3
2	40 Ti	20.0	21.9	21.7	−.2
2	40 Ti	20.0	21.9	21.9	.0
2	……	20.0	21.9	21.7	−.2
2	40 Ti	40.0	41.9	42.4	+.5
2	40 Ti	40.0	41.9	42.1	+.2
2	40 Ti	60.0	61.9	62.4	+.5
2	40 Ti	100.0	101.9	101.1	−.8

**Table 2:** Results of determinations of aluminum by the recommended procedure in the presence of possible interfering elements

Sample number^a^	Weight of sample	Other element added^b^	Aluminum			

			Added	Present	Found	Difference
						
	*g*	*mg*	*mg*	*mg*	*mg*	*mg*
169	2	35 Nb	20.0	21.9	22.1	+0.2
169	2	35 Nb	20.0	21.9	21.8	−.1
169	2	84 Nb	20.0	21.9	22.0	+.1
169	2	82 Mo	20.0	21.9	21.9	.0
169	2	1 P	20.0	21.9	21.9	.0
169	2	10 Sn^c^	20.0	21.9	22.1	+.2
169	2	10 Sn^d^	20.0	21.9	22.1	+.2
169	2	10 Sn^e^	20.0	21.9	21.8	−.1
169	2	10 V	20.0	21.9	22.0	+.1
169	2	119 W	20.0	21.9	21.9	.0
169	2	5 Zr	20.0	21.9	21.9	.0
168	2	………	20.0	20.4	20.3	−.1
168	2	………	20.0	20.4	20.7	+.3

a NBS Standard Sample. Standard Sample 168, Cr 20, Co 41, Ni 20, Mo 4, W 4, Nb 3, Ta 1.

b All samples contain an addition of 40 mg of Ti.

c Tin added as metal at start.

d Tin added to solution of ignited oxides.

e Tin added as solution to caustic filtrate.

**Table 3:** Results of determination of aluminum by the recommended procedure in high temperature alloys

NBS standard sample	Aluminum				No. of determinations	Wt of sample	Type of alloy
	Certificate value	Found	Difference	Range			
							
	%	%	%	%		*g*	
349	1.23 a	1.23	0.0	1.22 to 1.23	6	2	Ni 57, Cr 20, Co 14, Mo 4, Ti 3, A11.
1188	0.77 a	0.76	−.01	0.76 to 0. 77	3	2	Ni 73, Cr 15, Ti 2, Nb 1.
1189	1.20 a	1.17	−.03	1.17	3	2	Ni 73, Cr 20, Ti 2.5.
1191	1.55 a	1.54	−.01	1.51 to 1.55	8	2	Ni 55, Cr 19, Co 14, Mo 5, Ti 3.
1192	1.06 a	1.06	.0	1.06 to 1.07	5	2	Ni 57, Cr 18, Co 11, Mo 7, Ti 3.
106A	1.08	1.10	+.02	1.08	1	2	Nitriding steel.

a Provisional certificate values.

## Footnotes

1 Figures in brackets indicate the literature references at the end of this paper.

2 Diluted sulfuric acid (1+49) denotes 1 volume of concentrated sulfuric acid, sp gr 1.84, diluted with 49 volumes of water. If no dilution is specified, the concentrated analytical reagent is meant.

3 With cyanide present, all operations should be conducted in a hood.

4 A minimum addition of 25 ml of 8-hydroxyquinoline solution is recommended. In the presence of larger than usual amounts of aluminum or titanium, additional reagent may be required. For example, 40 ml of 8-hydroxyquinoline solution was used in the last experiment given in table 1.

5 The cyanide wash solution should be handled by air or bulb.

6 The use of “weight aliquoting” avoids the necessity of washing the precipitate and handling strongly alkaline solutions in contact with glassware. Weighing on a trip balance to the nearest half-gram or so provides adequate accuracy for the present application.

7 Each mg of aluminum in the aliquot requires approximately 0.22 ml of 8- hydroxyquinoline solution. A small excess, 2 ml, is added to minimize absorption of excess reagent by the precipitate.
